# Impact of the COVID-19 outbreak on the US equity sectors: Evidence from quantile return spillovers

**DOI:** 10.1186/s40854-021-00228-2

**Published:** 2021-03-02

**Authors:** Syed Jawad Hussain Shahzad, Elie Bouri, Ladislav Kristoufek, Tareq Saeed

**Affiliations:** 1 Montpellier Business School, University of Montpellier, Montpellier Research in Management, Montpellier, France; 2 South Ural State University, Chelyabins, Russian Federation; 3 Adnan Kassar School of Business, Lebanese American University, Beirut, Lebanon; 4 Institute of Information Theory and Automation, The Czech Academy of Sciences, Prague, Czech Republic; 5 Institute of Economic Studies, Faculty of Social Sciences, Charles University, Prague, Czech Republic; 6 Nonlinear Analysis and Applied Mathematics (NAAM)-Research Group, Department of Mathematics, Faculty of Science, King Abdulaziz University, Jeddah, Saudi Arabia

**Keywords:** Quantile return spillovers, US equity sector indices, COVID-19 outbreak, Granger causality, Global risk aversion

## Abstract

The aim of this study is to examine the extreme return spillovers among the US stock market sectors in the light of the COVID-19 outbreak. To this end, we extend the now-traditional Diebold-Yilmaz spillover index to the quantiles domain by building networks of generalized forecast error variance decomposition of a quantile vector autoregressive model specifically for extreme returns. Notably, we control for common movements by using the overall stock market index as a common factor for all sectors and uncover the effect of the COVID-19 outbreak on the dynamics of the network. The results show that the network structure and spillovers differ considerably with respect to the market state. During stable times, the network shows a nice sectoral clustering structure which, however, changes dramatically for both adverse and beneficial market conditions constituting a highly connected network structure. The pandemic period itself shows an interesting restructuring of the network as the dominant clusters become more tightly connected while the rest of the network remains well separated. The sectoral topology thus has not collapsed into a unified market during the pandemic.

## Introduction

The abrupt outbreak of coronavirus disease 2019 (COVID-19) and its quick spread throughout the world, especially the US, in early 2020 have cast the world into an unprecedented global health crisis. Declared a pandemic by the World Health Organization (WHO) on March 11, 2020, COVID-19 has been a major factor in hundreds of thousands of deaths, led to the suffering of millions of people, and put the lives of billions across more than 193 countries at risk.^1^

The COVID-19 outbreak is more than a health crisis, it is a catastrophic event that has led the global economy into delirium and, for this reason, will likely make it into (not only) economic history textbooks as the Great Lockdown recession. With its overwhelming magnitude, the COVID-19 outbreak represents one of the steepest few recessions since the Great Depression of the 1930s. For example, US GDP decreased by 5% from Q4 2019 to Q4 2020 due to the COVID-19 outbreak.^2^ Furthermore, the US unemployment rate spiked from 4.4% in March 2020 to 14.7% and 13.3% in April 2020 and May 2020, respectively.^3^ Due to increased uncertainty and consequent panic selling, global stock markets crashed. In the US, major equity benchmarks such as the Dow Jones Industrial Average experienced a 7.79% decline on March 9, 2020 and a 9.9% plunge on March 12, 2020, with the latter representing one of the worst downswings ever recorded in US history. The S&P 500 index faced similar heavy losses and its circuit breakers were triggered four times during March 2020. Besides its adverse impact on the aggregate indices, the real economic impact of the COVID-19 outbreak has spread to several equity sectors, triggering heavy losses especially in banks, energy and gas, industrials, and consumer discretionary such as airlines and travel. In banks and financial institutions, the spike in the share of non-performing loans coupled with the decrease in interest rate margins due to reductions in policy rates has crushed earnings and thus the share prices of banks and financial institutions. In fact, the valuations of banks and financial institutions fell by almost 39%, exceeding the loss experienced in the overall US market. In the energy and gas sector, the Great Lockdown recession has led to a large decrease in the global demand for energy, especially crude oil, which has squeezed the profit margins of energy and gas companies and led to a significant decrease in their stock prices. Spending on travel and leisure has been hit considerably by lockdowns and travel restrictions, while staples and survival supplies show positive purchase upticks.

In such an environment of heightened economic and financial uncertainty, during the extreme catastrophic event related to the COVID-19 lockdown, investors and portfolio managers are very keen to understand the transmission of shocks across US equity sectors for the sake of portfolio diversification and risk management.^4^ Generally, investors and portfolio managers switch between sector indices when making investment decisions. For example, during turnarounds in the economy, they move into defensive stocks which are considered recession-proof investments. During economic booms, cyclical stocks become an appealing destination given their high price sensitivity to business cycle fluctuations. In this regard, a good understanding of the origins and magnitude of shocks across equity sectors is essential to enhance the ability of market participants to achieve their main financial goals while minimizing risk. As such, informed investors can optimize their decision-making regarding portfolio diversification while considering the information transmission among equity sectors and the effect of the disaster risk related to COVID-19.

While several studies examine return spillovers across the US sector indices within the system, they often use mean-based measures of connectedness (e.g., Diebold and Yilmaz 2012), ignoring return spillovers at the tails of the return distribution. This represents a crucial shortcoming in the related literature as the sole focus on mean-based connectedness masks potential differences in the patterns and strength of information transmission across the lower, middle, and upper quantiles of return distributions, representing stress, tranquil, and bullish periods, respectively. As indicated in related studies, market linkages are stronger during bearish states than normal or bullish states (e.g., Aloui et al. 2020; Shahzad et al. 2018; Baumöhl and Shahzad 2019; Saeed et al. 2020). Accordingly, a tail-based connectedness analysis emerges as a suitable alternative approach given its ability to provide more comprehensive information on how return connectedness occurs among the US equity sector indices in extreme market conditions such as the COVID-19 outbreak period.

In light of the above discussion, the aim of this study is to examine the extreme return spillovers among the US stock market sectors during the COVID-19 outbreak. Using daily prices of 10 US equity sectors indices between September 20, 2017 and April 30, 2020, covering the catastrophic event of the COVID-19 outbreak, we apply a tail-based connectedness approach, extending the vector autoregression approach of Diebold and Yilmaz (2012) from the mean-based level to the quantile-based level (Saeed et al. 2020). Accordingly, our analyses capture the dynamics of return spillovers in a time-varying setting, not only on average but also across upper, middle, and lower quantiles. We conduct additional analysis involving linear and nonlinear Granger causality tests to relate the total return spillover index and spillover from an individual sector with risk aversion. A high level of return spillover among a US equity sector reflects systemic risk; consequently, risk aversion is expected to be higher during stress periods such as COVID-19 and thus a strong link is expected between the spillover index and risk aversion.

Our study contributes on several fronts. Firstly, we build on previous studies that consider return spillovers during stress periods across equity markets and equity sectors (e.g., Choudhry and Jayasekera 2014; Yarovaya et al. 2016), commodities and equity markets (Degiannakis et al. 2013; Zhu et al. 2016; Kang et al. 2017; Ma et al. 2019; Naeem et al. 2020), economic growth (Abosedra et al. 2020), or uncertainty and financial markets (You et al. 2017). With evidence of intensified return spillovers at the sector-level, especially at extreme lower quantiles and during catastrophic events such as COVID-19, our findings support and complement the existing sector-level evidence. Secondly, our analyses extend previous evidence on the spillovers among return shocks often conducted at the mean-based level by exploring them at the left tail of the return distribution (e.g., Shahzad et al. 2018; Baumöhl and Shahzad 2019; Saeed et al. 2020). Thirdly, we add to previous studies by showing evidence of Granger causality between risk aversion and spillovers. Specifically, our results show a bi-directional non-linear Granger causality between global risk aversion and return spillover among the US sectors (overall and in a majority of sectors), which is high during the COVID-19 outbreak. Notably, in the financial sector, the Granger causality runs from financials to the risk aversion index.

Our analyses provide interesting results related to the COVID-19 outbreak, stock market phases, and tail behaviour. They show evidence of intensified connectedness among the returns of US equity sectors around the COVID-19 outbreak, which reflects the rise in instability of the system of connectedness. Weak levels of return spillovers are reported among specific equity sectors, suggesting potential diversification benefits. Connectedness at the tails is stronger in general but there is no evidence of asymmetric tail dependency. Interestingly, network structural changes during COVID-19 are mostly within the bulk of the distribution which are reflected not only in the spillover connectedness increase but also a tighter relationship between selected sectors. However, the tail dependence does not change markedly during the outbreak as it has already been very high in general leading to an almost completely connected graph of dependencies among sectors. Further analysis involving linear and nonlinear Granger casualties shows that the total return spillover index and spillover from an individual US equity sector index are related to the risk aversion index. Overall, the results indicate changing spillover and network structures with respect to the market state, from a well-defined sectoral clustering during stable periods to a highly connected network structure during adverse and beneficial market conditions. Although the pandemic period is characterized by network restructuring as the dominant clusters become more tightly connected, the rest of the network remains well separated without apparent signs of a unified market collapse into a singularity during the critical period.

## Related studies

The existing literature generally examines the direction and strength of shock spillovers in the stock markets using conventional techniques based on rolling correlation coefficients (Brida and Risso 2010), GARCH-based processes (Liu et al. 2017), or Granger causality tests (Wang et al. 2017; Al-Yahyaee et al. 2019). However, these techniques do not allow us to uncover the spillovers within the overall network topological structure of the system of stock market returns and mostly consider mean-based spillovers only. In this regard, the network connectedness approach of Diebold and Yilmaz (2012) emerges as an effective instrument for measuring systemic risk in complex financial systems. It has been applied in recent finance and economic literature (e.g., Maghyereh et al. 2016; Yarovaya et al. 2016; Husain et al. 2019; Yoon et al. 2019), allowing us to uncover the dynamics of spillovers among financial series within a system. However, the approach of Diebold and Yilmaz (2012), which is based on forecast error variance decompositions from a generalized vector autoregressive (VAR) model, also relies on average-based estimators. Therefore, it is unable to capture extreme risk spillovers at the tails of the return distributions of financial series.

Evidence of extreme spillovers in the stock markets has been reported in the academic literature, especially during extreme events such as the global financial crisis of 2007–2008 (GFC). A first strand of literature considers inter-country stock market linkages at the aggregate levels and reveals evidence that the spillover effects across a large set of stock markets vary under different market conditions such as bearish, normal, and bullish (Shahzad et al. 2018). Aloui et al. (2020) examine the extreme linkages between the US and large emerging stock markets using copula functions and indicate time-variability in the linkages. Dendramis et al. (2015) show the stronger impact of large shocks compared to the impact of small shocks. Baumöhl and Shahzad (2019) use a quantile coherency approach across leading stock markets and find evidence of a tail dependence network that seems to intensify following the GFC. Londono (2019) focuses on the stock market indices of 32 developed and emerging economies and finds evidence of spillover of extreme events in both upper and lower quantiles.

A second limited strand of literature focuses on the spillovers within sectors. Billio et al. (2012) study the banking sector and show evidence of extreme spillover effects in bank performance. Dah and Fakih (2016) decompose gender wage differentials in the banking sector using quantile regression. Wang et al. (2017) apply Granger causality tests to the downside tail-risk measure of financial institutions and show that stress periods such as the GFC and the European debt crisis shape the structure and dynamics of the causality network. Tail risk spillovers among financial institutions are also examined by Foglia and Angelini (2020) who indicate the importance of each financial sector, especially banks as a main transmitter of risk. Deev and Lyócsa (2020) find evidence of stronger connectedness among financial institutions during stress periods. Using firm-level data, Aldieri et al. (2020) consider innovation and spillovers by examining the environmental knowledge spillovers on firms' productivity in Europe, Japan and the US.

The above literature review indicates the need for studies considering the extreme spillovers across equity sectors within network topological metrics to reveal the impact of the COVID-19 outbreak on the network of return spillovers. We address this important research gap within a quantile VAR approach of connectedness in both static and time-varying settings. In this regard, our analysis applies quantile-based methods to the US equity sector indices, which makes it comparable to the methods applied by recent studies (e.g., Shahzad et al. 2018; Baumöhl and Shahzad 2019), especially Saeed et al. (2020), although we differ in our focus on the effects of COVID-19 on the network of extreme connectedness of the US equity sector indices as well as including risk aversion within the analyses of extreme connectedness. Our work is closely related to recent research on the effects of COVID-19 within the literature on economics and finance. Notably, it nicely complements the growing literature on the effects of COVID-19 on financial markets (e.g., Alola et al. 2020; Aloui et al. 2020; Bouri et al. 2020; Shahzad et al. 2020; Sharif et al. 2020; Gupta et al. 2021), which has so far ignored the impact of this catastrophic event on extreme co-movements among the US equity sectors.

## Methods

First, the quantile generalized forecast error variance decomposition is applied to uncover the return spillovers among the US stock market sectors under various upper, middle, and lower quantiles, in light of the COVID-19 outbreak. Second, various Granger causality tests are used to examine the causal relationship between the global risk aversion index and the time-varying total spillover index and spillover indices from a particular sector to all remaining sectors.

### Quantile connectedness

#### The quantile factor VAR

The quantile connectedness methodology starts with the factor VAR model: $y_{t} = c + \mathop \sum \nolimits_{i = 1}^{p} B_{i} y_{t - i} + { }f_{t} + e_{t}$; where $f_{t}$ represents a common factor, that is the overall stock market index.

The n-variable quantile factor VAR estimated at the τ-th conditional quantile is:

1 $$y_{t} = c\left( \tau \right) + \mathop \sum \nolimits_{i = 1}^{p} B_{i} \left( \tau \right)y_{t - i} + { }\left( \tau \right)f_{t} + e_{t} \left( \tau \right), \tau \in \left( {0,1} \right)$$

where $y_{t}$ is the n-vector of stock sector returns at time *t*, $c\left( \tau \right)$ and $e_{t} \left( \tau \right)$ represent, respectively, n-vector of intercepts and residuals at quantile τ, $B_{i} \left( \tau \right)$ is the matrix of lagged stock sector returns at quantile τ, with *i* = 1,…, p, $f_{t}$ is a common factor, that is market index, and $\left( \tau \right)$ is its coefficient at quantile τ. $\hat{B}_{i} \left( \tau \right)$ and $\hat{c}\left( \tau \right)$ can be estimated while assuming that the residuals fulfill the population quantile restrictions, $Q_{\tau } (e_{t} \left( \tau \right)\left| {F_{t - 1} ) = 0} \right.$, where $F_{t - 1}$ denotes the information set available at time *t*-1. In fact, the population *τ*th conditional quantile of response *y* is:

2 $$Q_{\tau } (y_{t} |F_{t - 1} ) = \hat{c}\left( \tau \right) + \mathop \sum \nolimits_{i = 1}^{p} \hat{B}_{i} \left( \tau \right)y_{t - i } + \left( \tau \right)f_{t - i}$$

Equation (2) is estimated at every quantile (Cecchetti and Li 2008). Following Ando et al. (2018), the Diebold and Yilmaz (2012) model is extended, where the *m*(*m*—1) bilateral interactions among an *m*-vector of variables, $y_{t}$, are approximated by the h-step-head generalized forecast error variance decomposition (GFEVD) of an underlying VAR in $y_{t}$.

#### The quantile spillover indices

Equation (1) is rewritten as an infinite order vector moving average process:

3 $$y_{t} = \mu \left( \tau \right) + \mathop \sum \nolimits_{s = 0}^{\infty } A_{s} \left( \tau \right)e_{t - s} \left( \tau \right),\;t = 1, \ldots ,T$$

with,

$$\mu \left( \tau \right) = \left( {{\text{I}}_{n} - B_{1} \left( \tau \right) - \cdots - B_{p} \left( \tau \right)} \right)^{ - 1} c\left( \tau \right),A_{s} \left( \tau \right) = \left\{ {\begin{array}{*{20}l} {0,s < 0} \hfill \\ {I_{n} ,s = 0} \hfill \\ {B_{1} \left( \tau \right)A_{s - 1} \left( \tau \right) + \cdots + B_{p} \left( \tau \right)A_{s - p} \left( \tau \right),s > 0} \hfill \\ \end{array} } \right.$$

where $y_{t}$ is given by the sum of residuals $e_{t}$ at every quantile τ (i.e., $e_{t} \left( \tau \right))$.

The variance decomposition is conducted using orthogonal innovations while the VAR innovations are generally correlated contemporaneously. To overcome the variables ordering issue, we follow Diebold and Yilmaz (2012) and use the generalized VAR framework of Koop et al. (1996) and Pesaran and Shin (1998). Because shocks to each variable are not orthogonalized, the sum of contributions to the variance of forecast error is not necessarily equal to one. Hence, the GFEVD of a variable attributable to shocks of different variables for a forecast horizon *H* is computed as:

4 $$\theta_{ij}^{g} \left( H \right) = \frac{{\sigma_{jj}^{ - 1} \mathop \sum \nolimits_{h = 0}^{H - 1} \left( {e_{i}^{^{\prime}} A_{s} \sum e_{j} } \right)^{2} }}{{\mathop \sum \nolimits_{h = 0}^{H - 1} \left( {e_{i}^{^{\prime}} A_{s} \sum e_{j} } \right)}}$$

where $\theta_{ij}^{g} \left( H \right)$ denotes the contribution of the *j*th variable to the variance of the forecast error of the *i*th variable at horizon H, ∑ is the variance matrix of the vector of errors, ${\upsigma }_{jj}$ is the *j*th diagonal element of the Σ matrix, and ${\text{e}}_{i}$ is a vector with a value of 1 for the *i*th element and 0 otherwise.

Each entry of the variance decomposition matrix is scaled as follows:

5 $$\tilde{\theta }_{ij}^{g} \left( H \right) = \frac{{\theta_{ij}^{g} \left( H \right)}}{{\mathop \sum \nolimits_{j = 1}^{N} \theta_{ij}^{g} \left( H \right)}}$$

Using the quantile GFEVD, we define various measures of connectedness at the τth conditional quantile.

6 $$TSI\left( \tau \right) = \frac{{\mathop \sum \nolimits_{i = 1}^{N} \mathop \sum \nolimits_{j = 1,i \ne j}^{N} \tilde{\theta }_{ij}^{g} \left( \tau \right)}}{{\mathop \sum \nolimits_{i = 1}^{N} \mathop \sum \nolimits_{j = 1}^{N} \tilde{\theta }_{ij}^{g} \left( \tau \right)}} \times 100$$

7 $$S_{alli} \left( \tau \right) = \frac{{\mathop \sum \nolimits_{j = 1,i \ne j}^{N} \tilde{\theta }_{ij}^{g} \left( \tau \right)}}{{\mathop \sum \nolimits_{j = 1}^{N} \tilde{\theta }_{ij}^{g} \left( \tau \right)}} \times 100$$

8 $$S_{iall} \left( \tau \right) = \frac{{\mathop \sum \nolimits_{j = 1,i \ne j}^{N} \tilde{\theta }_{ji}^{g} \left( \tau \right)}}{{\mathop \sum \nolimits_{j = 1}^{N} \tilde{\theta }_{ji}^{g} \left( \tau \right)}} \times 100$$

9 $$NS_{i} \left( \tau \right) = S_{alli} \left( \tau \right) - S_{ialli} \left( \tau \right)$$

10 $$S_{ij} \left( \tau \right) = \tilde{\theta }_{ji}^{g} \left( \tau \right) - \tilde{\theta }_{ij}^{g} \left( \tau \right){ }$$

$TSI(\tau )$ is the total spillover index at quantile τ; ${S}_{alli}\left(\tau \right)$ is the directional spillover index from all indices to index *i* at quantile τ, which is denoted by “TO”; ${S}_{iall}(\tau )$ is the directional spillover index from index *i* to all stock indices at quantile τ, which is denoted by “FROM”; $N{S}_{i}\left(\tau \right)$ is the net total spillover index at quantile τ; and ${S}_{ij}$ is the pairwise spillover index at quantile τ.

The above quantile connectedness measures are estimated suing a VAR lag order of 1 (selected based on the Bayesian information criterion) and a 10-step-ahead GFEVD. To reflect the time variation in the spillover measures, a rolling-window approach is adopted (e.g., Shahzad et al. 2018; Saeed et al. 2020).

### Granger causality tests

The Taylor series approximation causality test is based on the Taylor expansion of the nonlinear function:

11 $$x_{t} = f^{*} \left( {x_{t - 1} , \ldots ,x_{t - q} ,y_{t - 1} , \ldots ,y_{t - n} ,\vartheta^{*} } \right) + \varepsilon_{t}$$

where $\vartheta^{*}$ is a vector, $x_{t}$ and $y_{t}$ are weakly stationary series, and $f^{*}$ is an unknown function but assumed to represent the causal relationship between $y_{t}$ and $x_{t}$. Moreover, for every point of the sample (parameter) space $\vartheta^{*} \in {\Theta }$, $f^{*}$ has a convergent Taylor expansion. To examine the non-causality hypothesis, i.e., $y_{t}$ does not cause $x_{t}$, we have:

12 $$x_{t} = f^{*} \left( {x_{t - 1} , \ldots ,x_{t - q} ,\vartheta } \right) + \varepsilon_{t}$$

We linearize $f^{*}$ and increase the function form into a $k^{th}$ order Taylor series around an arbitrary sample space. After the approximation and re-parametrization of ${f}^{*}$, we obtain:

13 $$x_{t} = \theta _{0} + \sum\nolimits_{{j = 1}}^{q} {\theta _{j} x_{{t - j}} + \sum\nolimits_{{j = 1}}^{n} {\gamma _{j} y_{{t - j}} } } + \sum\nolimits_{{j_{1} = 1}}^{q} {\sum\nolimits_{{j_{2} = j_{1} }}^{q} {\theta _{{j1j_{2} }} x_{{t - j_{1} }} x_{{t - j_{2} }} } } + \sum\nolimits_{{j_{1} = 1}}^{q} {\sum\nolimits_{{j_{2} = 1}}^{n} {\varphi _{{j_{1} j_{2} }} x_{{t - j_{1} }} y_{{t - j_{2} }} } + } \sum\nolimits_{{j_{1} = 1}}^{n} {\sum\nolimits_{{j_{2} = j_{1} }}^{n} {\gamma _{{j_{1} j_{2} }} y_{{j_{1} j_{2} }} y_{{t - j_{1} }} y_{{t - j_{2} }} } + \cdots } + \sum\nolimits_{{j_{1} - 1}}^{q} {\sum\nolimits_{{j_{2} = j_{2} }}^{q} \ldots + } \sum\nolimits_{{j_{k} = j_{k} - 1}}^{q} {\theta _{{j_{1} \ldots j_{k} }} x_{{t - j_{1} }} \ldots } x_{{t - j_{k} }} + \cdots + \theta _{{j_{1} j_{2} }} x_{{t - j_{1} }} x_{{t - j_{2} }} + \sum\nolimits_{{j_{1} = 1}}^{n} {\sum\nolimits_{{j_{2} = j_{1} }}^{n} \ldots } \sum\nolimits_{{j_{k} = j_{k} - 1}}^{n} {\gamma _{{j_{1} \ldots j_{k} }} y_{{t - j_{1} }} \ldots } y_{{t - j_{k} }} + \varepsilon _{j}^{*}$$

where $\varepsilon_{t}^{*} = \varepsilon_{t} + R_{t}^{\left( k \right)} \left( {y,x} \right),R_{t}^{\left( k \right)}$ represents the remainder with $n \le k$ and $q \le k$.

We use the principal components to overcome the multicollinearity and degrees of freedom problem and test the null hypothesis of zero coefficients of principal components as:

14 $$General = \frac{{\left( {SSR_{0} - SSR_{1} } \right)/p^{*} }}{{SSR_{1} /\left( {T - 1 - 2p^{*} } \right)}}$$

The problem of degree of freedom can be tackled by assuming that the general model is “semi-additive”:

15 $$x_{t} = f\left( {x_{t - 1} , \ldots ,x_{t - q} ,\vartheta_{f} ) + g(y_{t - 1} , \ldots ,y_{t - n} ,\vartheta_{g} } \right) + \varepsilon_{t}$$

where $\vartheta^{\prime} = \left( {\vartheta^{\prime}_{f} ,\vartheta^{\prime}_{g} } \right)^{^{\prime}}$ is the parameter vector.

If $g\left( {y_{t - 1} , \ldots ,y_{t - n} ,\vartheta_{g} } \right) = constant$, then $y_{t}$ does not Granger cause $x_{t}$. To obtain the statistic called *Additive*, we linearize both functions into $k^{th} - order$ Taylor series.

The ANN causality test uses a logistic function. The approximation of the equation $g\left( {y_{t - 1} , \ldots ,y_{t - n} ,\vartheta_{g} } \right)$ is obtained using:

16 $$\vartheta_{0} + \tilde{\mu }_{t}^{^{\prime}} \alpha + \mathop \sum \nolimits_{j = 1}^{p} B_{j} \frac{1}{{1 + e^{{ - \gamma_{j}^{^{\prime}} \mu_{t} }} }}$$

where $\vartheta_{0} \in R$, $\mu_{t} = \left( {1,\tilde{\mu }_{t}^{^{\prime}} } \right)^{^{\prime}}$ is a $\left( {n + 1} \right) \times 1$ vector, $\tilde{\mu }_{t} = \left( {y_{t - 1} , \ldots ,y_{t - n} } \right)^{^{\prime}}$, $\alpha = \left( {\alpha_{1} , \ldots ,\alpha_{n} } \right)^{^{\prime}}$ are $\left( {n \times 1} \right)$ vectors, and $\gamma_{j} = \left( {\gamma_{j0} , \ldots ,\gamma_{jn} } \right)^{^{\prime}}$ for $j = 1, \ldots ,p$, are $\left( {n + 1} \right) \times 1$ vectors. The null hypothesis of the test is $\left\{ {y_{t} } \right\}$ does not cause $\left\{ {x_{t} } \right\}$. The estimation of the ANN-based causality test serves as (1) comparative analysis for the Taylor-based nonlinear causality test, and (2) a robustness check. The use of nonlinear causality tests also helps minimize possible estimation errors, since we use estimated transmission measures. Additionally, we use the VAR stability tests to ensure the stationarity of residuals.

## Data and empirical results

Firstly, we present summary statistics and some preliminary tests for the data series under study. Secondly, we discuss the results of return spillovers for the full-sample period and the COVID-19 subperiod, which is done using network analysis at the extreme upper and extreme lower quantiles. Thirdly, we consider the results of return spillovers obtained from a rolling-window analysis to capture time-variability in the return spillovers, especially those related to the COVID-19 subperiod. Fourthly, we apply the pairwise Granger causality approach in its linear and nonlinear forms to show that the total return spillover index and spillover from an individual sector are related to risk aversion.

### Dataset

The dataset used in this study is extracted from Thomson Reuters DataStream International, covering the period October 29, 2001 to October 27, 2020, which includes the COVID-19 outbreak. It consists of daily indices of 10 US stock sectors (energy, financials, industrials, utilities, consumer staples, health care, information technology, materials, consumer discretionary, and real estate). Notably, logarithmic return series are used in the analysis, and the related summary statistics and preliminary tests are reported in Table 1. Among the sector indices, the information technology sector has the highest average return for the whole sample period, and the real estate sector has the highest volatile returns. All return series depart from the Gaussian distribution, with a high level of kurtosis and non-zero skewness. All return series are stationary as shown by the results of the augmented Dickey-Fuller (ADF 1979) unit root and Kwiatkowski et al. (KPSS 1992) stationarity test statistics. The combination of a leptokurtic distribution and stationarity makes the series ideal candidates for quantile-based interconnectedness analysis.

**Table 1:** Descriptive and statistical properties of returns series

Sectors	Symbols	Mean	SD	Skewness	Kurtosis	J-B	ADF	KPSS
Energy	EN	0.000	1.756	− 0.839	20.118	61,100.8***	− 76.96***	0.413
Financials	FIN	0.003	1.910	− 0.227	20.774	65,295.1***	− 33.44***	0.112
Industrials	IND	0.020	1.352	− 0.447	12.070	17,157.8***	− 74.73***	0.063
Utilities	UTL	0.015	1.236	− 0.043	18.492	49,573.0***	− 76.96***	0.093
Consumer Services	CS	0.023	0.906	− 0.168	16.750	39,074.5***	− 79.44***	0.060
Healthcare	HC	0.023	1.102	− 0.215	12.971	20,571.3***	− 76.39***	0.279
Information Technology	IT	0.037	1.507	− 0.109	11.008	13,253.9***	− 78.47***	0.331
Materials	MAT	0.023	1.520	− 0.434	11.449	14,900.7***	− 74.60***	0.024
Consumer Discretionary	CD	0.035	1.336	− 0.281	12.339	18,077.1***	− 74.47***	0.187
Real estate	RE	0.018	1.942	− 0.201	22.388	77,672.1***	− 85.47***	0.042
Overall stock market index	MKT	0.023	1.220	− 0.447	15.930	34,695.5***	− 80.16***	0.135

J–B stands for Jarque–Bera test of normality. ADF and KPSS test unit root and stationarity, respectively

^***^Indicates rejection of null hypothesis of normality and unit root at 1% level of significance

### Eliminating cross-section correlations with factors

**Fig. 1:**
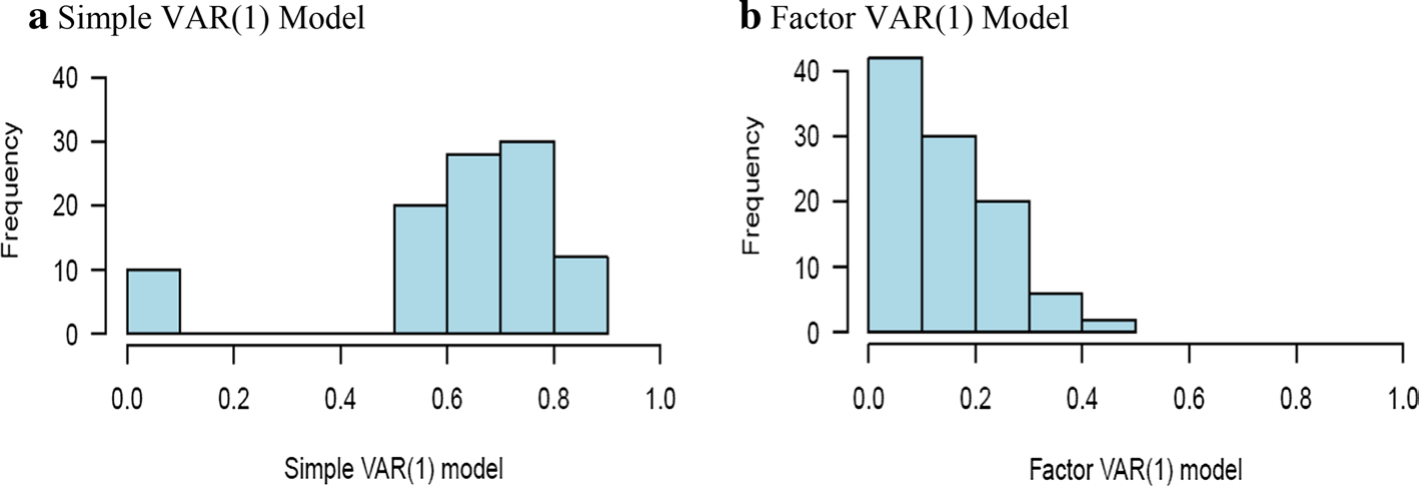
Comparison of absolute residual correlations, with and without factors. **a** Simple VAR(1) model. **b** Factor VAR(1) model. Note: The histograms show the distribution of the absolute pairwise correlations between the residuals of the simple VAR(1) model and our factor VAR(1) model evaluated by OLS

As the estimation of quantile regressions and thus also quantile connectedness can be problematic in a system of equations with cross-section correlations, we follow Ando et al. (2018) and remove the cross-section correlation in the system residuals by imposing a factor structure to the system. We use the overall stock market index as a common factor of the system. The optimal number of lags is selected with respect to the Bayesian information criterion and is identified as a single lag. Figure 1 shows the frequency distribution of the residual cross-correlations and reveals a marked difference between the simple VAR(1) and the factor VAR(1) models. The left panel shows that the original correlations mostly (around 90%) exceed 0.4, and more than 60% of the correlations are above 0.6. The right panel illustrates how these correlations are successfully covered by the common factor, as around 90% of the correlations are below 0.4, the majority of them below 0.2. This reduction is very close to that reported by Ando et al. (2018) and strongly supports the validity of the common factor used.

**Fig. 2:**
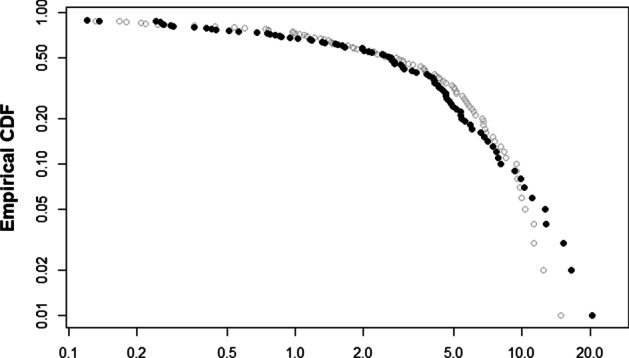
Spillover densities of VARs with and without factors. Notes: This figure shows the spillover densities of the models without factors (as a dashed grey line) and with factors (black circles)

This kind of factorization is likely to affect the network properties of the system. Following Greenwood-Nimmo et al. (2016), we present the spillover density of the system considered with and without the common factors in Fig. 2. Taking the steps of Acemoglu et al. (2012), the counter (complementary) cumulative distribution functions (CCDFs) are shown. The spillover density is normalized between 0 and 100 in accordance with Diebold and Yilmaz (Diebold and Yilmaz 2012) where higher values indicate stronger spillovers. We observe that for spillovers below approximately 10, which covers around 90% of the connectedness relationships, there is no visible difference between the original model and the factor model. However, for the highest spillovers, these are corrected by the factor model and pushed downwards. Should the common factors not be controlled for, these strongest estimated bilateral spillover effects would be overestimated. We thus continue with the factor VAR specification in the following analyses.

### Baseline network connectedness at the conditional mean and median

**Fig. 3:**
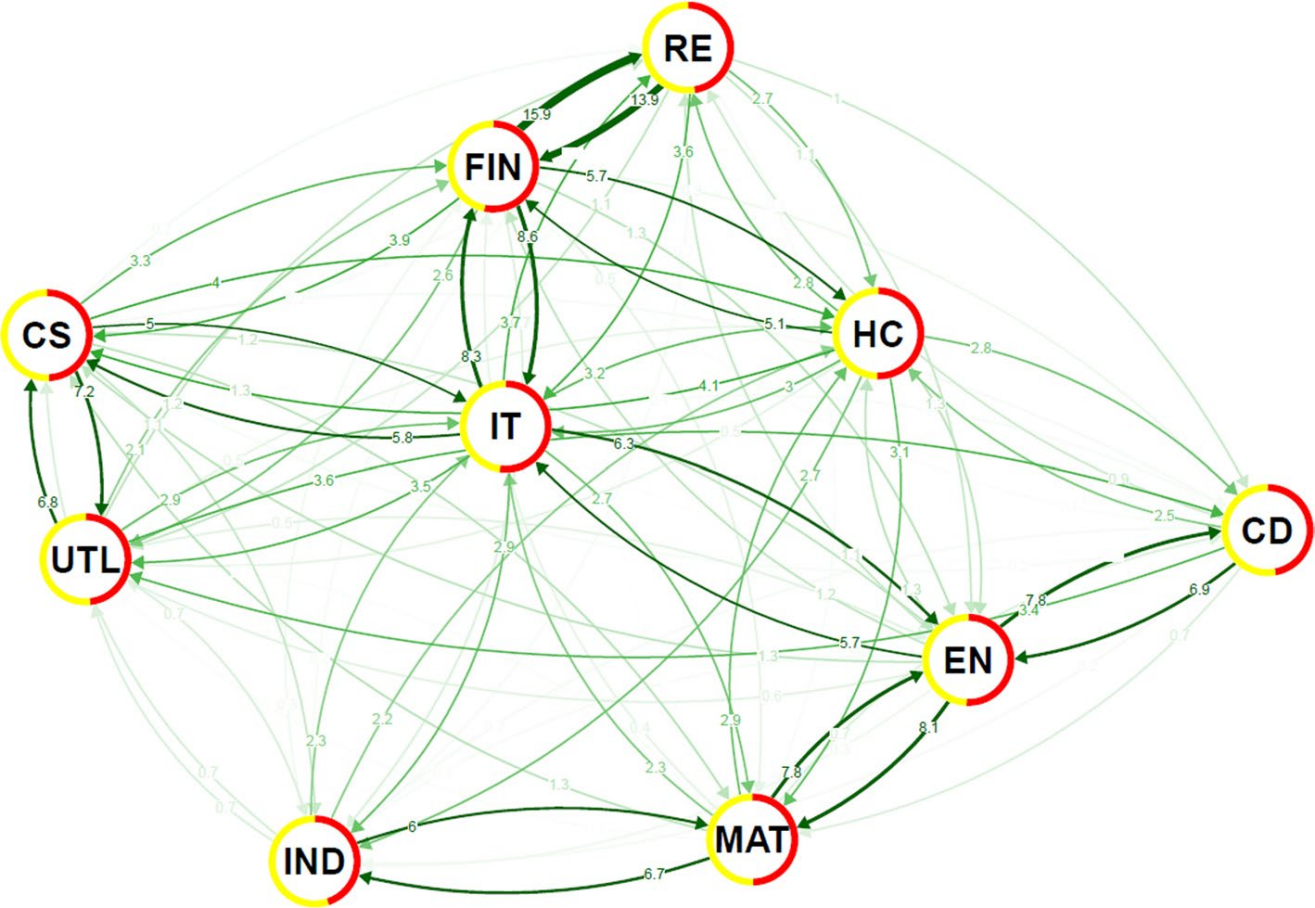
DY spillover based on conditional mean: ordinary least squares (OLS) estimator. Note: This network graph shows the degree of connectedness among the US sectors over the full sample period. The pair-wise connectedness is measured using the Diebold-Yilmaz framework. The thickness of an edge is proportional to its weight and spillover is shown as a numeric value on the start of an edge. Edges are drawn as curves and the direction of pairwise spillovers is shown through arrows. The net spillover for a node (US stock sector) is shown as pie around border where red is for transmission and yellow for reception. A higher percentage of red indicates that the node is a net spillover transmitter. The layout of the nodes is determined by applying the force-directed algorithm of Fruchterman and Reingold (1991)

**Fig. 4:**
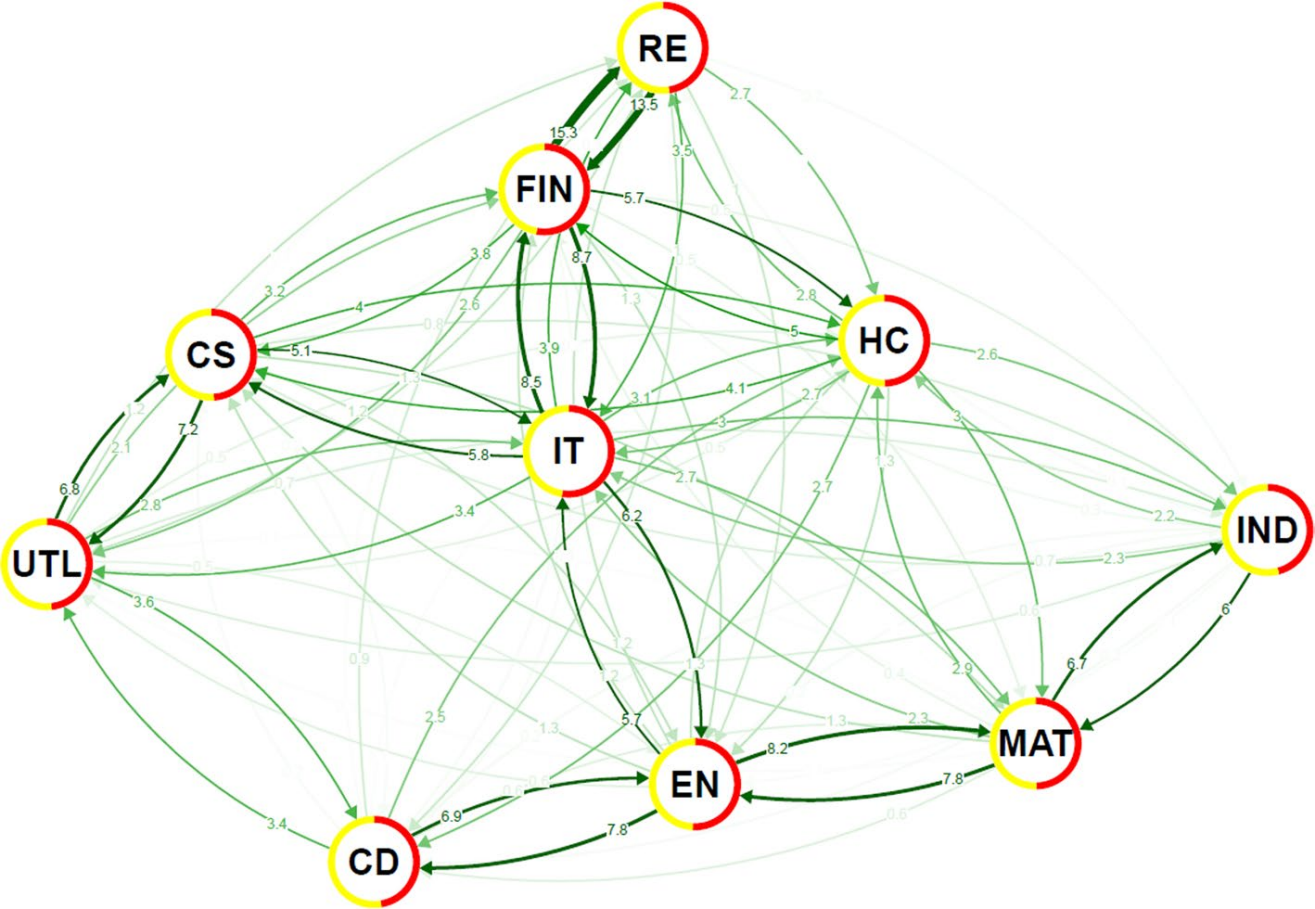
DY spillover based on conditional median: least absolute deviations (LAD) estimator. Note. See notes to Fig. 3

As a baseline case of the system topology, we inspect the network properties and connectedness at the conditional mean (estimated by OLS) and at the conditional median (estimated by LAD—the least absolute deviations estimator). Note that the former is based practically on an average effect over all possible quantiles, whereas the latter presents the results at the median, i.e. the 50th quantile. Comparison of these topologies can be seen as a first indication of quantile connectedness differences. Figures 3 and 4 show the network structure of the OLS and LAD estimators, respectively. Most features are shared across the two methods indicating that there is either only weak quantile dependence of the spillovers or such dependence is only present for a small part of the distribution, most likely the extreme quantiles, i.e. very low and/or the very high. In both figures, we see that there are three groups of sectors with strong connections: (i) real estate, financials and information technology, (ii) energy, basic material and industrial, and (iii) consumer staple and utilities. The weakest pair is of the health care and consumer discretionary. The connecting sector in the network turns out to be the information technology sector and this is again true for both methods.

### Network connectedness with respect to the shock size

**Fig. 5:**
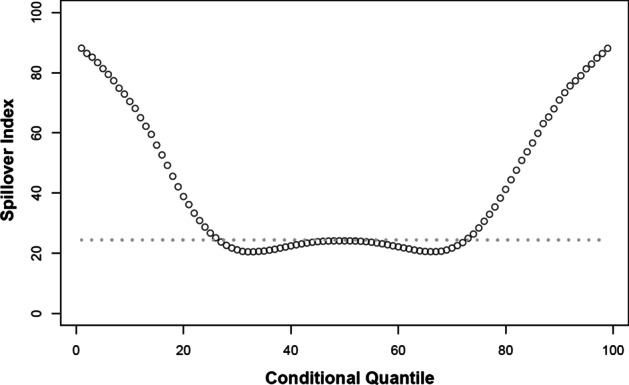
Variation in the DY spillover index over the conditional distribution. Notes: This figure shows the values of the spillover index defined in (6) evaluated at the $\tau$ th conditional quantile (plotted as a circle) relative to the value at the conditional mean (shown by the dashed line)

When studying the possible quantile dependence in the financial markets, be it correlations, regression, or, as in our case, spillovers, we are primarily interested in the actual existence of the quantile dependence of these measures and secondarily in the (a)symmetry of this quantile dependence. Figure 5 presents the variation in the spillovers over the quantile range. We see that for roughly the three central quintiles, i.e. the central 60% of the distribution, the spillover values are close to the conditional mean values (dashed line) slightly below 30%. For the quantiles below 0.2 and above 0.8, we observe sharp increases in the spillover values. For the most extreme cases, i.e. below quantiles 0.05 and above 0.95, the spillover index jumps to close to 90%. Note that the spillover dependence on quantiles is mostly symmetrical suggesting that large adverse shocks spill over on a similar scale as large beneficial shocks. This is consistent with results reported for stock markets as well as forex and commodity markets (Barunik et al. 2016, 2017; Ando et al. 2018; Barunik and Kocenda 2019).

**Fig. 6:**
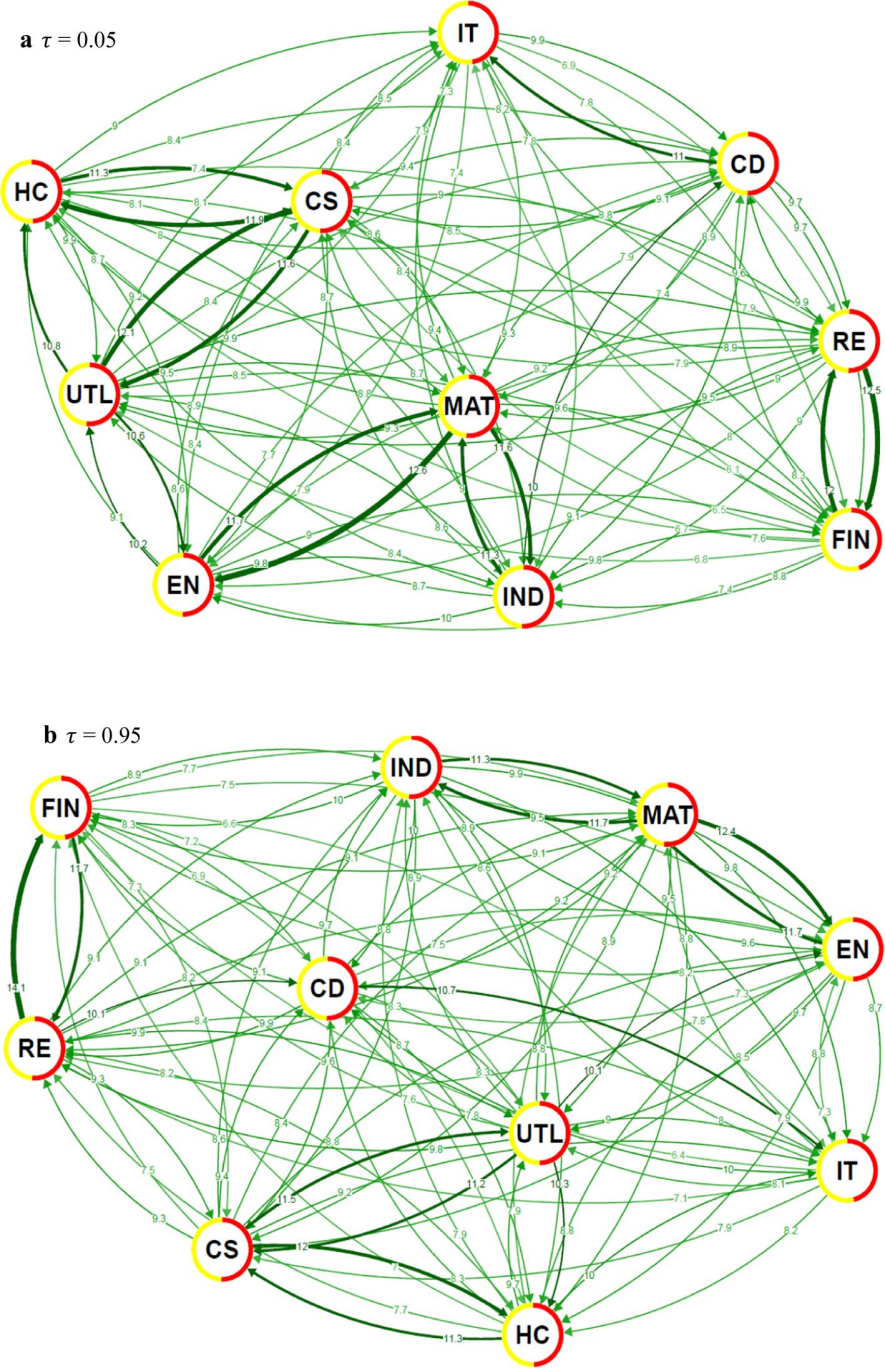
Network visualizations for quantile level spillovers at lower ($\tau$ = 0.05) and upper ($\tau$ = 0.95) tails of the return distributions. **a** $\tau$ = 0.05, **b** $\tau$ = 0.95. Note. See notes to Fig. 3

The detailed inspection of the network spillover structure in the case of extreme events, as shown in Fig. 6, reveals a structural change in the system topology. For standard-size events around the distribution bulk, we see several well-defined clusters of sectors. However, for the extreme cases, such topology vanishes and an almost completely connected graph emerges. In other words, the dominant role of the information technology sector disappears during extreme times and all sectors show high values of spillovers. This is true for both adverse and beneficial shocks. There is no clear difference between the two, neither topology-wise, nor spillover-size-wise. Combined with the fact that the sectoral network is built on the factor-filtered data, we can say that these extreme events (both positive and negative) are mostly exogenous and driven by unexpected situations not covered or sufficiently reflected by the factors.

### Temporal dynamics of the connectedness and tail-dependence structure

We have already shown that the connectedness between the US sectors materializing in the network topology of sectoral stock returns possesses a profound non-linearity reflected in much stronger spillovers in the tails of the underlying distributions, i.e. during extreme events. To further investigate the temporal stability of such dynamics, we inspect the dependence using a sliding window of 200 trading days.

**Fig. 7:**
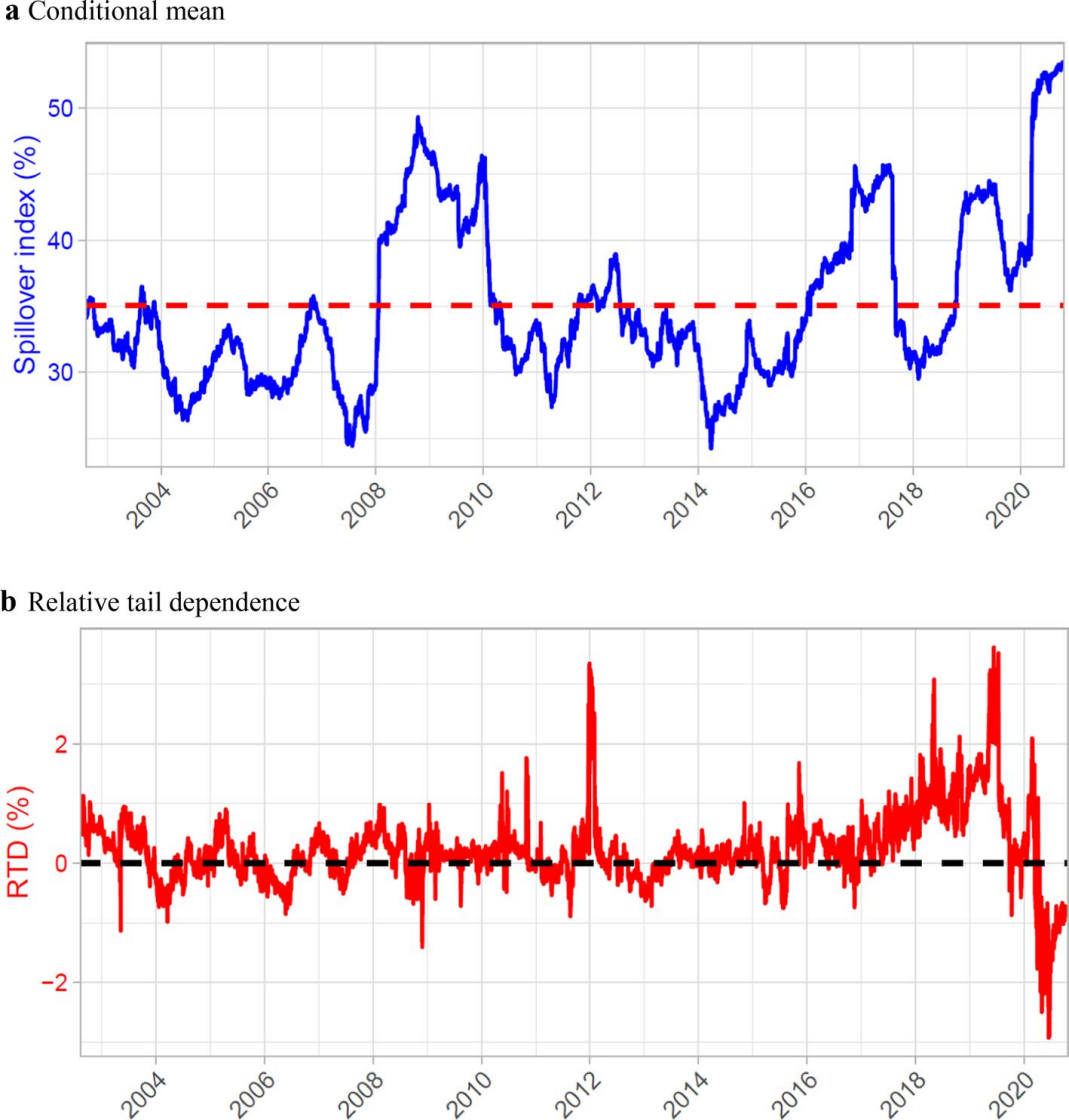
Time-varying spillover and relative tail dependence. **a** Conditional mean, **b** relative tail dependence. Notes: This figure shows the value of the spillover index defined in (6) evaluated at the mean (shown as the blue line in panel (**a**)) and the difference between the spillover index at the 95th and 5th conditional quantiles (shown as the red line in panel (**b**)). The results are obtained from rolling regressions with a window length of 200 trading days, lag order of 1 day and forecast time horizon of 12 days. The dates shown correspond to the last day of each rolling window

Figure 7 shows the dynamics of the overall spillover level of the system for the conditional mean and relative tail dependence index (the difference between two extreme quantile spillovers i.e., 95% and 5%). The overall picture reveals that the spillover index follows a sharply increasing trend during the GFC period (2008–09) and the start of the COVID19 pandemic in the first quarter of 2020; the index fluctuates between 40 and 50%. The current COVID-19 period shows a rapid spike, above 50%, even for the conditional mean. The relative tail dependence shown in panel (b) of the figure is the difference in the positive–negative tail nexus. The dynamics represent the symmetry of the spillovers for the positive and negative periods as most differences remain within the 2% band, compared for example to Ando et al. (2018), in which important events are reflected in differences above 10%, or Barunik and Kocenda (2019) and Barunik et al. (2017) who show confidence intervals at around 2.5% and 5% of the deviation, respectively.

**Fig. 8:**
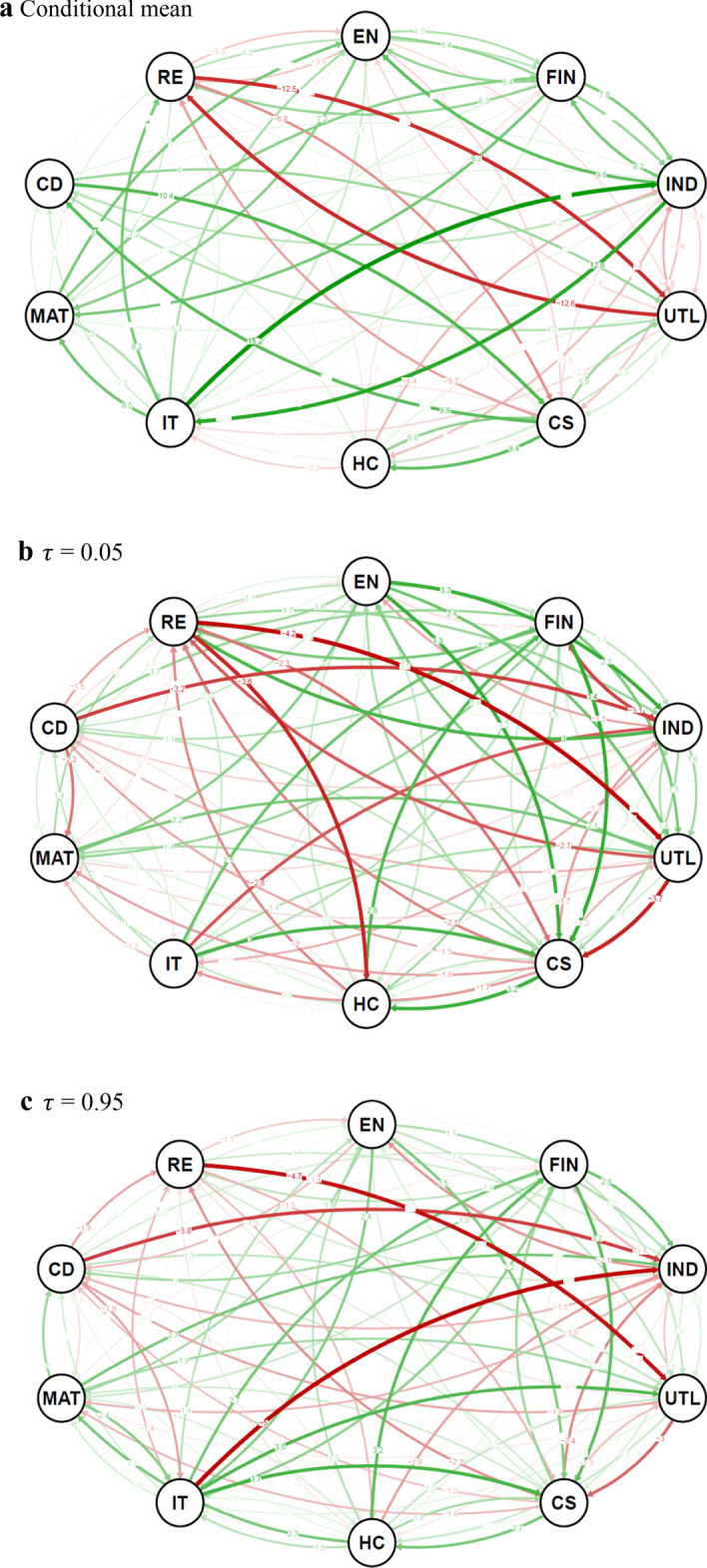
Change in spillover for pre and post COVID19 sub-sample. **a** Conditional mean, **b** $\tau$ = 0.05. **c** $\tau$ = 0.95, Note: These graphs of US sector networks show the difference in connectedness in a system that consists of the US sectors for the pre- and during COVID19 sub-sample periods (each sub-sample contains 204 days). The connectedness is measured through the spillover approach proposed by Diebold-Yilmaz (2012). The thickness of an edge is proportional to its weight while colour implies increase (green) and decrease (red) in spillover during the COVID-19 pandemic

**Fig. 9:**
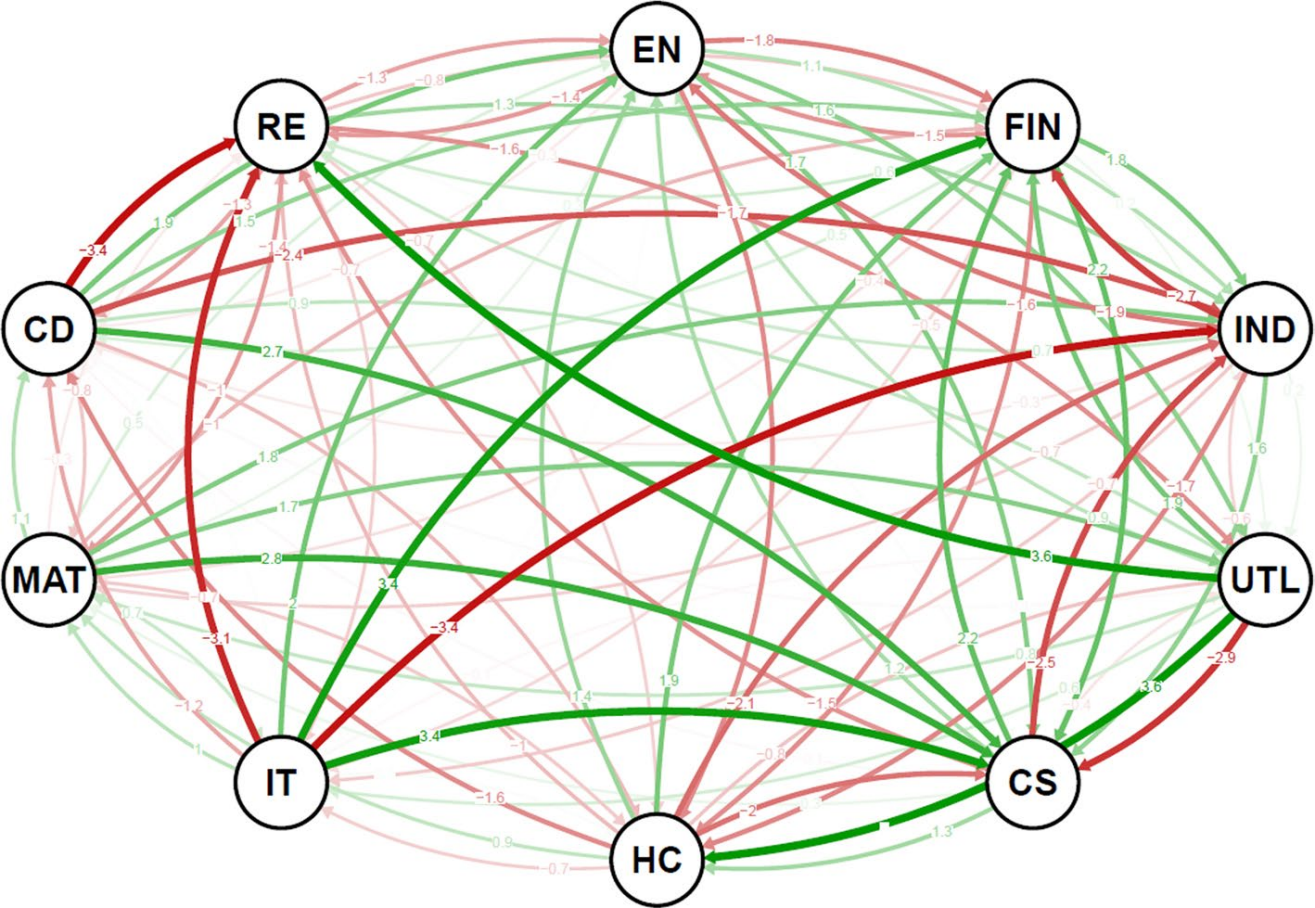
Difference in spillover between lower ($\tau$ = 0.05) and upper ($\tau$ = 0.95) tails during COVID19 sub-sample. Note: This graph of the US sector network shows the difference in connectedness in the system for the lower and upper quantile levels during the COVID-19 sub-sample period. The connectedness is measured through the spillover approach proposed by Diebold-Yilmaz (2012). The thickness of an edge is proportional to its weight while colour implies increase (green) and decrease (red) in spillover during COVID-19 pandemic

As the COVID-19 period seems to have imprinted itself mostly in spillovers at the distribution bulk, which might be due to a rather swift resurgence of the stock market after the monetary injection, we look closely at whether the network topology has also changed. Figures 8, 9 shows the differences in the spillover index in the network for the bulk of the distribution as well as for extreme events. We see that the network’s core tightens, as represented by the green links in the charts. Interestingly, we see the largest gains in the spillover index between clusters rather than within them. Most clusters come closer during the pandemic period. The connection between the industrial and information technology sectors is the one that tightens the most, bringing the industrial sector closer to the network’s centre. The medium-term perspective on the interconnectedness structures thus pays off, as recent studies (Mazur et al. 2020) suggest a much stronger system restructuring. From the other side, there are several connections that weaken (shown in red in the charts), most profoundly the connection between the utilities and real estate sectors. The real estate sector thus remains quite detached from the network, keeping its strong connection to the financial sector but otherwise quite independent. The utilities sector, in a sense, follows the position of the real estate as its connections to other sectors mostly decrease and even the connection to its cluster member (the consumer services sector) is not strengthened. This separation of the real estate sector is even more profound at the extremes where we observe a decreasing spillover index from and to this sector for almost all pairs. Similar dynamics are found for the other renegade of the original structure—the utilities sector—which further detaches from the consumer services sector to which it was most tightly connected before the pandemic. Overall, the evidence for the extreme events is more mixed than for the bulk of the distribution which is, however, not surprising as the overall relative tail dependence (Fig. 7) does not vary much so that the spillover gains are balanced by losses. Nevertheless, financial and consumer services become clear information receivers for extreme cases, which nicely corresponds with the effect of the pandemic on these two sectors. This clearly highlights how different the current crisis is from the global financial crisis a decade ago when negative events cascaded from the financial sector to the rest of the financial markets as well as the whole economy. Back then, negative news came from the financial sector. Now, the financial sector, in a sense, waits and reacts to how the situation develops. This goes against the results of Akhtaruzzaman et al. (2020) who find that financial institutions play a central role based on their dynamic cross-correlations analysis. However, their analysis covers only the very beginning of the pandemic (March 2020) which illustrates only the very first reactions of the financial markets before the actual economic policies started taking effect and the markets could react more reasonably to the new situation. In retrospect, during the 2007–2008 financial crisis in the USA, Hippler et al. (2019) find that financial institutions in fact played a central role when negative news was connected to the financial world, whereas they reacted more mildly when negative news came from the real economy. As suggested by Wang et al. (2017), there might be a change of network topology within the financial sector during such critical times. However, such detailed study must be left for future research as it goes a level deeper than our sectoral examination. For policymakers, this suggests that the current pandemic-induced economic downturn is mostly a real economy issue, so potential government interventions should be directed towards the real economy sectors that have been hit by restrictions. The financial market thus well reflects what we observe not only in the USA but also globally, as (mostly small) businesses and services have a hard time coping with the situation while financial and real estate markets function and prosper quite well. Such a development, should it take longer than a few months, might lead to a more pronounced restructuralization of the real economy as companies and firms with higher operating flexibility are more likely to succeed during the pandemic than companies with larger fixed assets (Liu et al. 2020).

From a big picture perspective, the network has changed structurally during the COVID-19 critical period. To see whether this specific situation, likely leading to global risk aversion (Yue et al. 2020), has a more general influence on spillovers levels, we apply Granger causality tests between a global risk aversion (GRA) index and return spillovers.

### Granger Causality between global risk aversion and return spillovers

The results presented to this point suggest that there might be connection between market state or distress and the way information spills over among sectors. To test such an assertion, we set the benchmark by the standard Granger (1969) causality test built on vector autoregression and complement it with two tests of nonlinear causality, specifically those using the Taylor series approximation and artificial neural networks, following Péguin-Feissolle and Teräsvirta (1999) and Albulescu et al. (2019). The nonlinearity tests are a natural extension of the standard causality tests as we have already shown that the interconnectedness is quantile-dependent, hence nonlinear. As a proxy of a market state, we use the global risk aversion (GRA) metric recently constructed by Bekaert et al. (2019).^5^ This is a time-varying daily index that reflects the risk appetite computed from observable financial information at high frequencies.

We are interested in possible Granger causality between the spillover indices (overall spillover index and spillover from a specific sector to all other sectors) and the GRA index.^6^ Table 2 reports the results of linear and non-linear Granger causality tests. First, we notice a bidirectional linear causality between the overall spillover among US equity sectors and the risk aversion, which suggests that the market uncertainty has led to structural changes in the connectedness properties (abbreviated as SOI, spillover index, in the table). Interestingly, the statistical significance of the causal flow from overall spillover to GRA is more pronounced when using the nonlinear tests, indicating that the overall spillover among US equity sectors affects GRA. This finding suggests that an increase in the risk spillover among equity sectors leads to an increase in the GRA level (which is measured using instrument like bond and equity market spreads and realized variances) and vice versa. At sectoral level, we find that the GRA index Granger causes spillovers from all sectors to others, except energy and basic material sectors. On the other hand, the GRA index is impacted by all sector level spillovers, except in industrial sector.

**Table 2:** Granger causality between global risk aversion (GRA) and spillover measures

	GRA $\to$ SOI			SOI $\to$ GRA			Overall conclusion
	Linear	Taylor	ANN	Linear	Taylor	ANN	
Overall	87.77***	2.364	1.231	11.711***	45.626***	133.91***	GRA $\leftrightarrow$ SOI
	[0.000]	[0.124]	[0.351]	[0.000]	[0.000]	[0.000]	
*From sector TO all others*							
EN	2.076	0.823	3.139	4.355**	79.747***	34.592***	GRA $\leftarrow$ SOI
	[0.125]	[0.438]	[0.581]	[0.012]	[0.000]	[0.000]	
FIN	21.390***	3.995**	26.091***	15.435***	434.47***	155.09***	GRA $\leftrightarrow$ SOI
	[0.000]	[0.018]	[0.000]	[0.000]	[0.000]	[0.000]	
IND	17.650***	5.801***	30.112***	0.429	1.265	1.680	GRA $\to$ SOI
	[0.000]	[0.003]	[0.000]	[0.651]	[0.201]	[0.169]	
UTL	125.17***	3.101**	3.827***	0.176	10.025***	7.273***	GRA $\leftrightarrow$ SOI
	[0.000]	[0.045]	[0.009]	[0.838]	[0.000]	[0.000]	
CS	133.64***	5.376***	74.733***	12.293***	19.744***	31.343***	GRA $\leftrightarrow$ SOI
	[0.000]	[0.004]	[0.000]	[0.000]	[0.000]	[0.000]	
HC	7.528***	12.153***	98.338***	7.995***	81.907***	62.312***	GRA $\leftrightarrow$ SOI
	[0.000]	[0.000]	[0.000]	[0.000]	[0.000]	[0.000]	
IT	10.216***	1.490	91.605***	1.751	8.827***	47.025***	GRA $\leftrightarrow$ SOI
	[0.000]	[0.225]	[0.000]	[0.173]	[0.000]	[0.000]	
MAT	0.304	0.145	0.304	3.310**	75.410***	26.225***	GRA $\leftarrow$ SOI
	[0.737]	[0.864]	[0.732]	[0.036]	[0.000]	[0.000]	
CD	10.296***	1.507	29.234***	13.886***	347.07***	115.74***	GRA $\leftrightarrow$ SOI
	[0.000]	[0.221]	[0.000]	[0.000]	[0.000]	[0.000]	
RE	7.096***	1.410	12.557***	4.538**	70.977***	33.143***	GRA $\leftrightarrow$ SOI
	[0.000]	[0.244]	[0.000]	[0.011]	[0.000]	[0.000]	

The above table displays the results of the linear, Tor-based and ANN-based nonlinear causality tests

Overall, the relationships between global risk aversion and the spillovers are not linear. The energy and material sectors are not driven by global risk aversion. The role of fear and uncertainty in the dynamic connectedness structure of financial markets is highlighted by recent studies (e.g., Bouri et al. 2020; Sharif et al. 2020; Gupta et al. 2021) showing that the dynamics are non-linear and strongly pronounced during the COVID-19 pandemic. These recent findings also concord with evidence put forward by Hesse and Frank (2009) linking funding stress and equity markets in advanced economies during the financial crisis. Our findings thus fit well into this perspective and extend the existing literature by examining the role of global risk aversion. In fact, the results of Granger causality tests can be explained considering previous evidence that risk aversion and sentiment dynamics are significant contributing factors of risky assets like stock prices, especially during stress periods such as the COVID-19 outbreak, which implies an interaction between risk aversion and stock indices. The risk aversion index can reflect the main changes in market conditions as evidenced from its peak during the 2008 global financial crisis whereas the risk aversion index declined following the reduction in the level of interest by the US Federal Reserve (e.g., Cipollini et al. 2018). In response to the COVID-19 pandemic, the US Federal Reserve has also pursued a loose monetary policy, which has intensified the interaction between loose monetary policy and the risk appetite of stock investors.

## Conclusion

Even though the coming of future economic and financial turmoil has been an important topic of expert as well as popular public discussion in the last few years, the pandemic of COVID-19 has come as an unexpected blast to the tightened economic reality. The pandemic is a quite different spark to ignite a global economic and financial crisis compared to other historical crises caused by mostly economic, technological, social or political effects (mortgages, complex financial instruments, oil, and technological bubbles to name a few). Even though the origin of the pandemic can be seen as mostly exogenous, which makes it unique compared to the other recent crises, its spread is inherent and endogenous to the ever-more interconnected and intertwined global economy. Still, its unambiguous distinction from the other economic critical events makes it attractive for a detailed analysis of its effects on the functioning of financial markets and how these differ from more standard, economically or specifically business cycle induced crises.

We provide a detailed, network-based interconnectedness study that focuses on the dynamics in tails of the distribution, i.e., during the critical periods the market is undergoing. By focusing on the sectoral structure of the US stock market, we reveal several interesting findings. Firstly, controlling for a common factor mostly affects the highest connections and spillovers. Omitting this filtration would lead to overrepresentation of the strong links in the system and not only would this bias the interpretation of the systemic structural changes, it would also misrepresent the split between systematic and idiosyncratic risk in the sectoral network leading to inefficient portfolio signals. Secondly, we show that the network structure of sectoral links is well-established with three rather tightly connected clusters, with the IT sector set as a central hub of the network. Thirdly, the overall sectoral spillovers increase markedly for extreme events and market conditions. In addition, the network topology changes its structure from well-defined clusters to an almost completely connected graph. The sector-driven dynamics of the network thus disappears during extreme market movements and the sectors tend to move together strongly. The tail dependence of the spillovers during extreme market conditions is symmetric, i.e., we do not find a significant difference between adverse and beneficial market conditions. Fourthly, the temporal connectedness is rather stable both in the bulk and the tails of the distribution, even though we find a mildly increasing trend in connectedness in the bulk and a mildly decreasing trend in the tails, suggesting a slow convergence. This is further reflected in the topology reorganization during the COVID-19 pandemic which, however, does not copy the general behaviour for the bulk versus tails network structure. We do not observe an implosion of the topology into a fully connected graph, but rather find more strongly connected original clusters, with real estate clearly detaching from the network. The financial sector also shows an interesting change in dynamics as it becomes a clear information receiver rather than a leader, as observed during the financial crisis of a decade ago, which only highlights the different roots of the last two episodes of global financial and economic turmoil. Fifthly, we find that changes in perception of market uncertainty, as reflected in global risk aversion, lead to structural changes in the sectoral network topology, both in calm and extreme market states. From the other side, the network topology changes only due to extreme events leading to changes in global risk aversion, making the relationship asymmetric.

Overall, the sectoral network structure of the US stock market has several intuitive features such as well-defined clusters with logical links of similar members (sectors) and an almost collapsed structure, in the network topology sense, during critical times. Our main contribution lies in revealing that the COVID-19-induced market turmoil stands out from other critical events in the market as there is a clear market reaction and a sectoral restructuring in the network connections but the structural changes occur mostly in the bulk of the distribution rather than the tails.

Our results open several interesting avenues for future research. Firstly, a study of high-frequency data would reveal the structural changes in more detail. Reactions to announcements of the pandemic spreading could be traced with higher precision. Secondly, focusing on the reaction of the connections, and the network structure in general, to monetary stimuli would reveal the efficiency of such policies. In addition, such an analysis could focus on similar policies in the preceding crises and how these decisions affected the market topology and its fragility or stability overall. As the network properties of the financial markets come to light as crucial characteristics of the system, moving from systematic to systemic risk, they remain highly relevant during the lockdown recession.

Although our analysis reveals some important insights into the quantile return spillovers across sectors in the US stock market which is the largest and most appealing market for global stock investors, it disregards quantile spillovers among sectors returns in other leading stock markets in Europe and Asia. This limitation can be addressed in future studies to see whether the results revealed in the US stock market during the COVID-19 outbreak hold in other stock markets. Some studies (Kou et al. 2014, 2020; Wen et al. 2019) apply interesting methods that can be the subject of future research.

## Data Availability

The datasets used and/or analyzed during the current study can be provided on reasonable request.

## Footnotes

1 https://healthwise.punchng.com/covid-19-global-death-toll-hits-177822-as-over-2571880-cases-declared-in-193-countries/

2 https://www.statista.com/statistics/188185/percent-chance-from-preceding-period-in-real-gdp-in-the-us/

3 https://www.statista.com/statistics/273909/seasonally-adjusted-monthly-unemployment-rate-in-the-us/

4 Since late March 2020, the US has had more cases of COVID-19 than any other country. As of June 2020, the US recorded more than two million cases, while the global number of cases exceeded 10 million.

5 Daily data on the GRA index are extracted from https://www.nancyxu.net/risk-aversion-index.

6 Note that the correlation among spillover measures is not substantially high and thus multicollinearity is not a concern. The correlation matrix is reported in the paper but is available upon request.
